# Traditional Herbal Plants and their Phytoconstituents Based Remedies for Respiratory Diseases: A Review

**DOI:** 10.2174/0118743064341009241210045737

**Published:** 2025-02-12

**Authors:** Luca Campbell

**Affiliations:** 1 Department of Science, Lovejoy High School, Lucas, Texas, TX, USA

**Keywords:** Pulmonary diseases, Obstructive lung diseases, Sarcoidosis, Restrictive lung diseases, Infectious lung diseases, Malignant lung diseases

## Abstract

Despite medical science advancements in recent years, pulmonary diseases are still hard to control and can be potentially life-threatening. These include asthma, COPD, lung cancer, cystic fibrosis, pneumonia, pleurisy, and sarcoidosis. These illnesses often cause severe breathing problems, which can be fatal if not treated properly. While some chemical drugs are used to treat these conditions, they can cause side effects and are not always effective.

Herbal medicine offers an alternative treatment option with fewer side effects and has shown promise in treating respiratory issues. Certain medicinal plants, such as garlic (Allium sativum), hawthorn (Crataegus rhipidophylla), moringa (Moringa oleifera), and ashwagandha (Withania somnifera), may help manage lung diseases. Natural compounds found in plants, like apple polyphenol, ligustrazine, salidroside, resveratrol, and quercetin, can also help reduce symptoms. These plants and compounds work by reducing cell overgrowth, fighting oxidative stress, lowering inflammation, stopping tumor growth, improving blood flow, and relaxing the airways.

This review outlines the types of plants and compounds that can be utilized in treating pulmonary conditions, along with their respective mechanisms of action.

## 1 INTRODUCTION

**Fig. (1):**
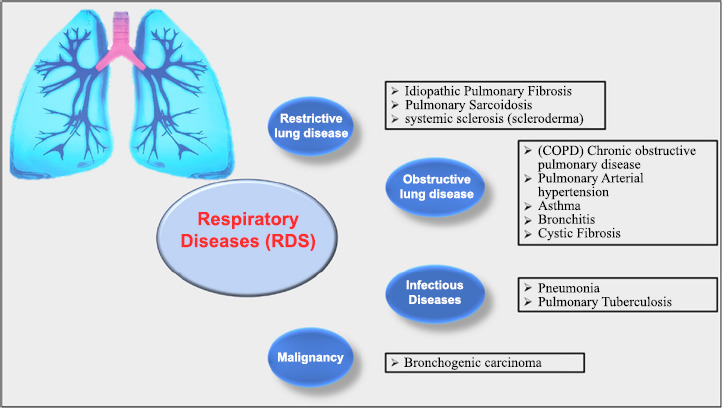
Schematic flow chart of respiratory diseases (RDs).

Chronic respiratory diseases are a major global health issue because harmful substances from the environment, as well as certain infections, can be easily inhaled. These diseases include chronic obstructive pulmonary disease (COPD), asthma, interstitial lung disease, pulmonary sarcoidosis, and pneumoconiosis, such as silicosis and asbestosis [1, 2]. Unfortunately, these conditions do not receive as much public attention or research funding as other diseases such as heart disease, cancer, stroke, diabetes, or Alzheimer’s. In addition, to better manage these illnesses, it is essential to understand how common they are, their impact on health, and their death rates, both globally and in specific areas. Respiratory infections, which can range from mild colds to life-threatening conditions, are one of the top reasons people visit doctors worldwide. These infections not only harm people’s quality of life but also create significant financial challenges for individuals and healthcare systems [3, 4] (Fig. **1**).

### 1.1 Respiratory Diseases: Origins and their Medicinal Cures

#### 1.1.1 Restrictive Lung Diseases (RLDs)

Restrictive lung diseases (RLDs) are a group of over 100 conditions that cause scarring in the lungs [5, 6]. Common symptoms include coughing, trouble breathing, reduced lung capacity, changes in chest X-rays, and inflammation observed in biopsies. RLDs can be identified as different disorders based on specific signs. For example, sarcoidosis involves inflammation without lung scarring, while idiopathic pulmonary fibrosis (IPF) is marked by severe scarring and lung damage [7, 8]. Diagnosing RLDs can be tricky because of their complexity, which makes it hard to measure how common they are. However, we know that RLDs affect adults worldwide [9, 10]. IPF is the most common type, with about 3 million cases globally and 130,000 in the U.S [11, 12]. It mainly affects older adults over 65, while sarcoidosis is more common in African Americans and Northern Europeans [13-15].

RLDs affect people differently—some recover over time, while others worsen, sometimes leading to death. IPF, a chronic scarring condition, has a poor prognosis, with most patients living only 2-5 years after diagnosis [16, 17]. The disease progresses differently for everyone; some experience slow changes, while others face rapid declines or fatal flare-ups. Sarcoidosis can range from mild, temporary symptoms to long-term health issues [18]. Diagnosing RLDs is difficult because they may overlap with other conditions or show no symptoms. For example, it takes an average of 2.1 years to diagnose IPF [17].

Most RLDs are classified as idiopathic, meaning the cause is unknown [19]. However, some cases are linked to systemic diseases (like rheumatoid arthritis), infections, or environmental factors (like smoking or asbestos) [1]. Genetic factors may also play a role—for example, mutations in certain genes can cause conditions like Hermansky-Pudlak syndrome [20, 21]. While the exact causes are unclear, age, lung injuries, and chronic inflammation seem to contribute. Inflammation and scarring involve immune cells (like macrophages and lymphocytes) and the buildup of proteins like collagen in the lungs [22]. In IPF, scarring is driven by lung cell damage, fibroblast growth, and the deposition of these proteins. Researchers are still trying to understand the origins of these fibroblasts and why symptoms vary so much between patients.

##### 1.1.1.1 Idiopathic Pulmonary Fibrosis (IPF)

Idiopathic pulmonary fibrosis (IPF) is a long-term lung disease that causes scarring in the lung tissue, making it harder to breathe over time [23]. It gets worse at different rates for different people—some experience slow changes, while others decline quickly. People with IPF are encouraged to take steps to avoid the sudden worsening of their condition. Eventually, the disease can lead to respiratory failure, which means the lungs can no longer work properly, and this is often fatal.

**Fig. (2):**
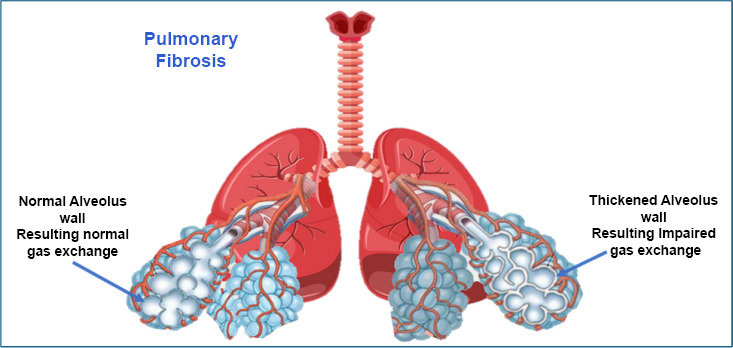
Schematics and overview of pulmonary fibrosis.

The disease involves the buildup of scar tissue in the lungs, which can go unnoticed for years before symptoms appear [24, 25]. When diagnosed, the lung damage often includes injury to the small air sacs (alveoli), overgrowth of certain lung cells, and increased scarring. Risk factors for IPF include smoking, exposure to certain dusts, infections, genetic changes, and aging [26] (Fig. **2**).

Pulmonary fibrosis can develop due to various risk factors, including genetics and repeated small injuries to the alveoli (tiny air sacs in the lungs). These injuries thicken the walls of the alveoli and cause abnormal cell behavior [27, 28]. Additionally, certain lung cells grow in unusual ways, worsening the disease. Since the body struggles to repair the damaged tissue, the healing process becomes chronic and inflamed. High levels of inflammation markers like IL-1 and TNF-α lead to ongoing problems with tissue repair and remodeling [29, 30].

Certain natural compounds, called phytoconstituents, may help manage these issues. For example, Pippali has antioxidant and immune-boosting effects, while Cordyceps sinensis reduces lung inflammation and irritation. Astragaloside may help with fibrosis and inflammation, and Salviae Miltiorrhizae can treat fibrosis caused by drugs like bleomycin. Isoliensinine reduces lung inflammation by lowering specific harmful substances in lung tissue. Triptolides have anti-inflammatory effects, and curcumin acts as an antioxidant and anti-inflammatory agent. In a study using a bleomycin-induced lung fibrosis model, curcumin reduced lung damage by lowering harmful proteins (like TGF-β1) and inflammation markers at multiple molecular levels [31, 32].

#### 1.1.2 Pulmonary Sarcoidosis

Sarcoidosis is a disease that can affect many parts of the body, like the skin, kidneys, heart, and nervous system, but it mostly impacts the lungs [33]. In the lungs, it can cause problems such as reduced lung function, lung scarring (pulmonary fibrosis), and the formation of small clumps of cells called granulomas [34]. Scientists are not entirely sure what causes sarcoidosis, but they think it happens when certain genes and inflammation caused by infections or irritants work together to form granulomas [33, 35]. Research also suggests that tiny particles called extracellular vesicles from the lungs might help spread the disease to other parts of the body.

One study found that fluid from the lungs of sarcoidosis patients contains certain proteins and high levels of Vitamin D and its binding protein. These findings suggest that materials from the lungs can enter the bloodstream, potentially spreading the disease [36].

Sarcoidosis affects the immune system and can seriously lower a patient’s quality of life. The exact process of how it develops is very complicated, which has made it hard to improve diagnosis and treatment. However, recent research is helping us understand the role of infections, genetic factors, inflammation, and environmental triggers in the development of the disease [37, 38]. Scientists are also studying plant-based drugs that might help treat lung sarcoidosis.

**Fig. (3):**
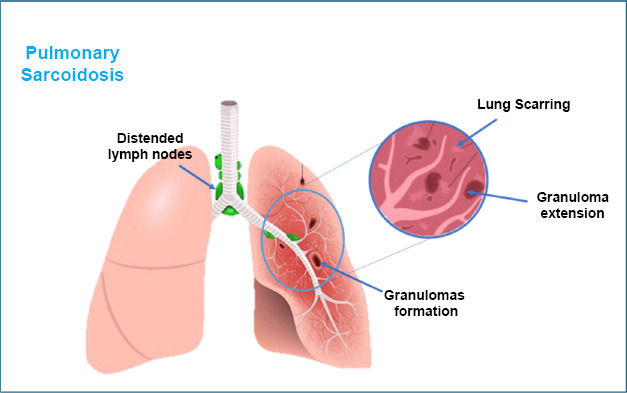
Pulmonary sarcoidosis: a scarred granulomas formation irritation in the lungs.

Moreover, because we do not fully understand sarcoidosis, diagnosing it can be tricky. Some patients are misdiagnosed (for example, with tuberculosis) or diagnosed too late, which can lead to permanent organ damage or even life-threatening complications. These issues can severely impact a patient’s ability to work, live comfortably, and maintain good health. Additionally, studying the underlying causes and molecular mechanisms of sarcoidosis is essential. Better understanding could lead to improved diagnosis and treatment options, offering better outcomes for patients. Some past studies have explored how the immune system responds to triggers like infections or certain proteins, leading to inflammation and damage in the lungs and other organs [39, 40] (Fig. **3**).

Some medicinal plants may help in treating sarcoidosis. Plants like Tulsi (Holy Basil), Amalaki (Indian Gooseberry), Haridra (Turmeric), Shatavari, Gokshura, Ashwagandha, and Guggul have useful properties, such as reducing inflammation, fighting infections, balancing the immune system, and preventing abnormal blood vessel growth. In Ayurvedic medicine, remedies like Punarnava Mandur and Kanchnar Guggul are known to support the immune system and reduce pain and inflammation.

One study tested a herbal medicine called Reuma herb on mice with sarcoidosis. Reuma herb is made from a mix of plant extracts, including Harpagophytum (Devil's Claw), Echinacea, and Filipendula (Meadowsweet). The study found that it could reduce inflammation and slow disease progression. These natural treatments show potential for managing sarcoidosis and could work alongside conventional medical approaches [41-43].

#### 1.1.3 Systematic Sclerosis

Systemic sclerosis (SSc) is a condition that affects the whole body, caused by an overactive immune system. It damages small blood vessels and disrupts the normal function of the immune system, certain cells called fibroblasts, and the process of scar tissue formation (called fibrosis). The exact cause of SSc is still unknown. Since the disease affects people in different ways, it can be tough for both doctors and patients to manage.

SSc does not just affect the skin, making it appear different; it can also harm organs like the lungs. The progression of the disease varies, but organ damage typically begins early. Unfortunately, people with SSc are 2.5 times more likely to die than healthy individuals, and this statistic has not improved in the last 40 years [44].

Fibrosis, or the buildup of scar tissue, is a key feature of SSc. It impacts not just the skin but also many internal organs. As a result, SSc is known as a disease that causes widespread scarring. Over the past 20 years, scarring in the lungs (called lung fibrosis) has been the leading cause of death in SSc patients [45].

**Fig. (4):**
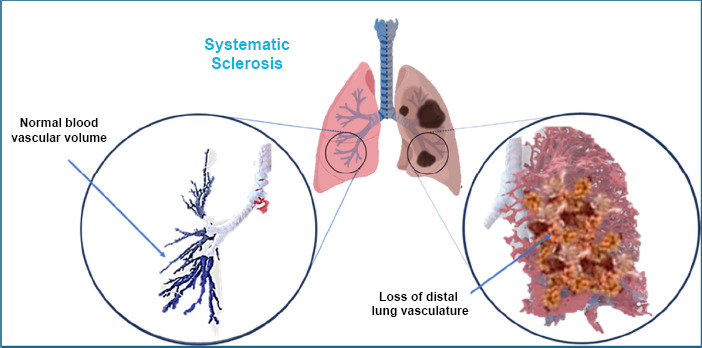
Graphical representation of systematic sclerosis.

Sadly, there are not many treatments that can stop or reverse the scarring. The FDA has approved only two drugs for SSc, but these mainly slow down lung damage rather than cure it [46]. Since the scarring continues, SSc remains incurable, and for some people, a lung transplant is the only option (Fig. **4**).

The way systemic sclerosis (SSc) causes lung scarring is very complex and not completely understood yet. It involves many different processes, with various molecules and cells playing a role. Understanding these processes and how they work together is key to finding new treatments.

Certain natural remedies might help treat SSc. For example, Capparis spinosa, Ginkgo biloba, and a mix of herbs called Gui-Zhi-Fu-Ling-Wan (GFW), which includes ingredients like cinnamon, peony species, and peach extracts, show potential [47, 48]. Other plants like evening primrose (Oenothera biennis), thunder god vine (Tripterygium wilfordii), and herbs like licorice (Glycyrrhiza uralensis), angelica (Angelica sinensis), and citrus (Citrus aurantium) are also commonly used for SSc treatment. These natural options could help reduce symptoms or slow the disease [49, 50].

### 1.2 Obstructive Lung Diseases

People with obstructive lung disease experience shortness of breath. Their lungs may be damaged, or their airways may be too narrow, which slows down how air leaves their lungs. Even after exhaling, some air gets trapped inside. This makes it harder to breathe, especially during physical activity. When they breathe quickly, they do not have enough time to fully exhale before the next breath.

Common causes of obstructive lung diseases include:

#### 1.2.1 Chronic Obstructive Pulmonary Disease (COPD)

COPD, or chronic obstructive pulmonary disease, is a group of lung conditions that get worse over time. These include emphysema, chronic bronchitis, damage to small airways, and long-term asthma. One of the main signs of COPD is feeling out of breath during everyday tasks, and this tends to get worse as time goes on [51]. People with severe COPD or asthma often have sudden worsening of symptoms, called flare-ups, which may lead to frequent trips to the emergency room.

Smoking, or even being a former smoker, is a major cause of COPD. It can damage the tiny air sacs in the lungs (emphysema) or lead to extra mucus and swelling in the airways (chronic bronchitis) [52, 53]. These problems make it harder for air to move in and out of the lungs, reducing the oxygen in the blood and potentially harming other organs [54].

Other common symptoms of COPD include a constant cough (with or without mucus), feeling tired, wheezing, and a tight feeling in the chest. These symptoms are sometimes mistaken as just part of aging. In some cases, people might not notice any symptoms until the disease has become quite severe.

**Fig. (5):**
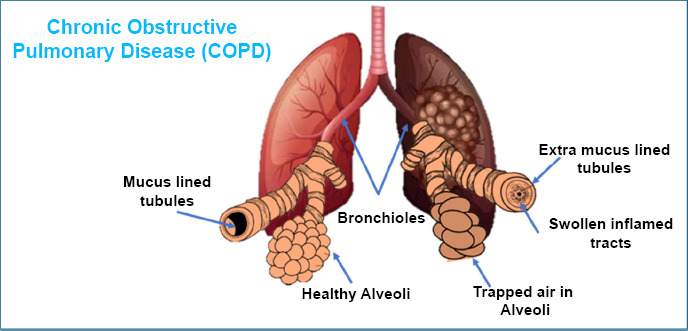
COPD: mucus-filled bronchus with air-filled alveoli.

Despite years of research, there is no way to repair damaged lungs or fully restore lung function. COPD is a condition that keeps getting worse over time, especially as people get older (Fig. **5**).

Researchers have looked into how herbal remedies can help with COPD. For example, Guo and colleagues tested a herbal product called Dansen on rabbits with emphysema and found it had a positive effect [55]. It was also safe when tested on dogs at a high dose. Another herbal treatment, Shengdi, was effective in reducing respiratory inflammation in rats. Based on these results, these therapies may be safer and more effective for COPD [56].

The extract of Thymus vulgaris (thyme) was found to help relax airways, suggesting it could be useful for asthma and COPD [57, 58]. A combination of thyme and primrose root significantly reduced bronchitis symptoms in patients with acute bronchitis and was both safe and effective [59]. Another study tested a blend of herbs, including garlic, horehound, boneset, aniseed, fennel, licorice, and hyssop, on horses with a condition similar to COPD. The treatment improved their breathing and kept them stable [55, 60].

Several other plants, such as Acacia leucophloea, Chelidonium majus, Datura stramonium, and Ephedra sinica, have also shown benefits for lung diseases with fewer side effects compared to conventional drugs. These findings suggest that medicinal plants might be safer and better alternatives for treating COPD [61, 62].

#### 1.2.2 Pulmonary Arterial Hypertension

Pulmonary hypertension (PH) is a long-term, slowly progressing condition that affects the pulmonary arteries in the lungs. It can be inherited but is also a major cause of death worldwide. People with PH often feel very tired, short of breath, experience chest pain or palpitations, and may suffer from anxiety, depression, swelling (edema), and frequent respiratory infections [63]. Without treatment, the average survival time for someone with PH is about two years [64].

PH causes damage to the arteries in the lungs, including inflammation, low oxygen levels, and abnormal cell growth in the artery walls, which makes it hard for damaged cells to die naturally [65]. These changes reduce oxygen flow to the lungs and increase pressure in the right side of the heart, which has to work harder. Over time, this leads to the thickening of the heart’s right ventricle (right ventricular hypertrophy) and disrupts normal oxygen and carbon dioxide exchange in the blood [66].

**Fig. (6):**
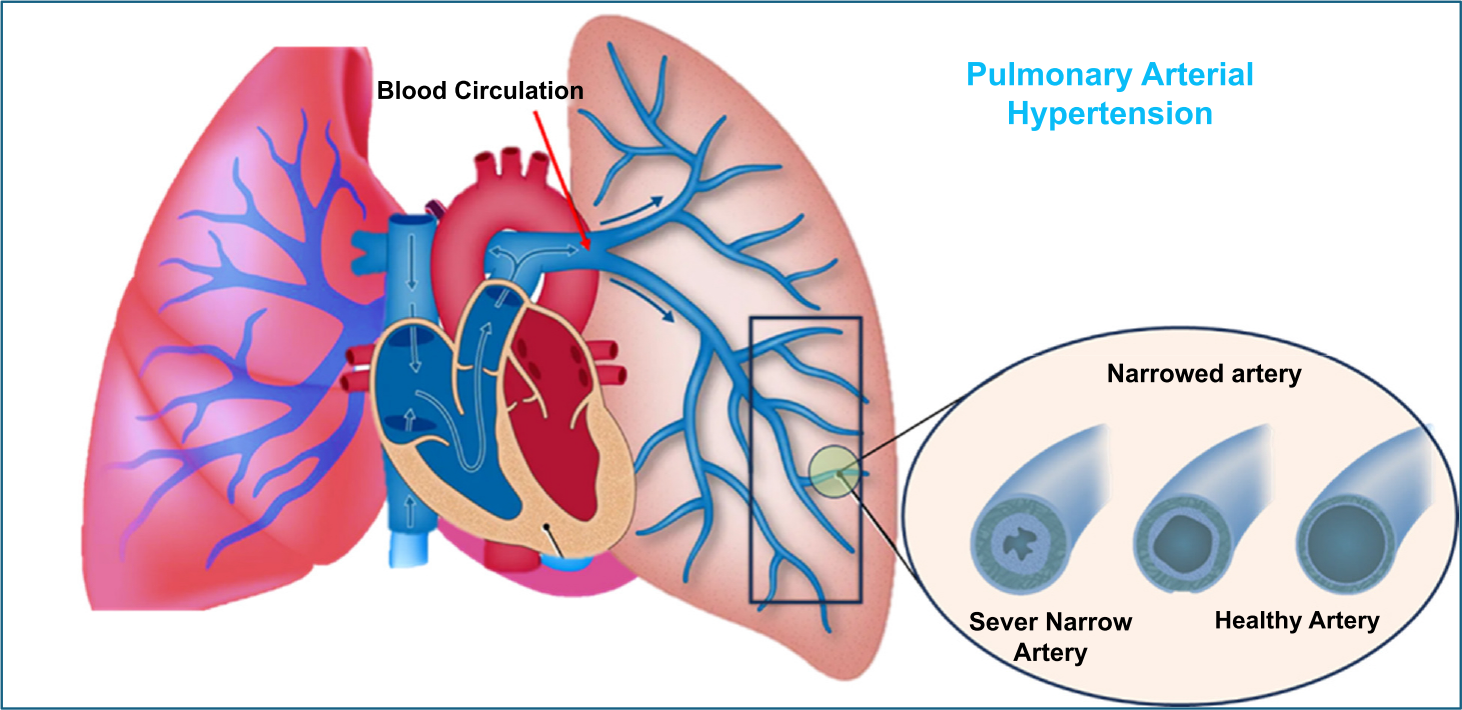
Narrow airways representation in pulmonary arterial hypertension.

Pulmonary arterial hypertension (PAH) is a specific type of PH and one of the most common. PAH can happen on its own or be caused by conditions like autoimmune diseases, drug use (*e.g*., amphetamines, fenfluramine), and certain toxins. Other types of PH are linked to lung diseases such as chronic obstructive pulmonary disease (COPD), low oxygen levels, blood clots, or inflammation from conditions like sarcoidosis [67] (Fig. **6**).

Scientists have studied pulmonary hypertension a lot, especially in developing effective medicines, but many of the drugs they have tested do not work very well [68]. As a result, researchers are exploring other molecules that might have a stronger impact on different lung diseases. In the past, ancient cultures used natural compounds like flavonoids, terpenoids, and isoflavonoids, along with herbal plants such as Clerodendrum Serratum, Crinum glaucum, Eclipta alba, Cnidium monnieri, Ficus bengalensis, Hemidesmus indicus, and Amburana cearensis. These plants were believed to help improve blood flow in the lungs, easing symptoms of pulmonary arterial hypertension [69-71].

#### 1.2.3 Asthma

Asthma is a condition where the airways become inflamed, making it hard to breathe. It is becoming more common in industrialized areas worldwide, currently affecting nearly 300 million people. By 2025, experts predict this number could rise to 400 million [72]. Since the 1970s, asthma cases have been increasing globally.

**Fig. (7):**
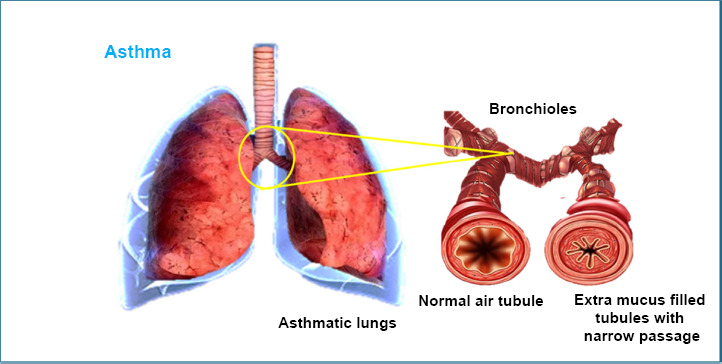
Graphics of asthmatic lung with mucus-filled narrow bronchioles.

In asthma, the immune system produces higher levels of a substance called IgE, which triggers inflammation. This causes the body to release chemicals like histamines, making the airways swell even more [73, 74]. For example, in Brazil, 24% of children and 20% of teenagers are affected by asthma. Over the past decade, doctors around the world have been prescribing anti-inflammatory treatments to reduce swelling in the airways and cut down on mucus buildup [75] (Fig. **7**).

Current asthma treatments are not working very well, therefore, researchers are looking into plants as a new option. Many modern medicines already come from plants, and herbal remedies for asthma might be a good choice because they usually have fewer side effects. Some plants that could help with asthma include *Ageratum conyzoides*, *Asystasia gangetica*, *Bacopa monnieri*, *Cassia sophera*, *Casuarina equisetifolia*, *Clerodendrum serratum*, *Cnidium monnieri*, *Crinum glaucum*, *Curculigo orchioides*, *Eclipta alba*, *Ficus bengalensis*, *Hemidesmus indicus*, *Amburana cearensis*, *Lepidium sativum*, *Mentha spicata*, *Momordica dioica*, and *Mucuna pruriens*. These plants and their relatives show promise for treating asthma naturally [76-78].

#### 1.2.4 Bronchitis

**Fig. (8):**
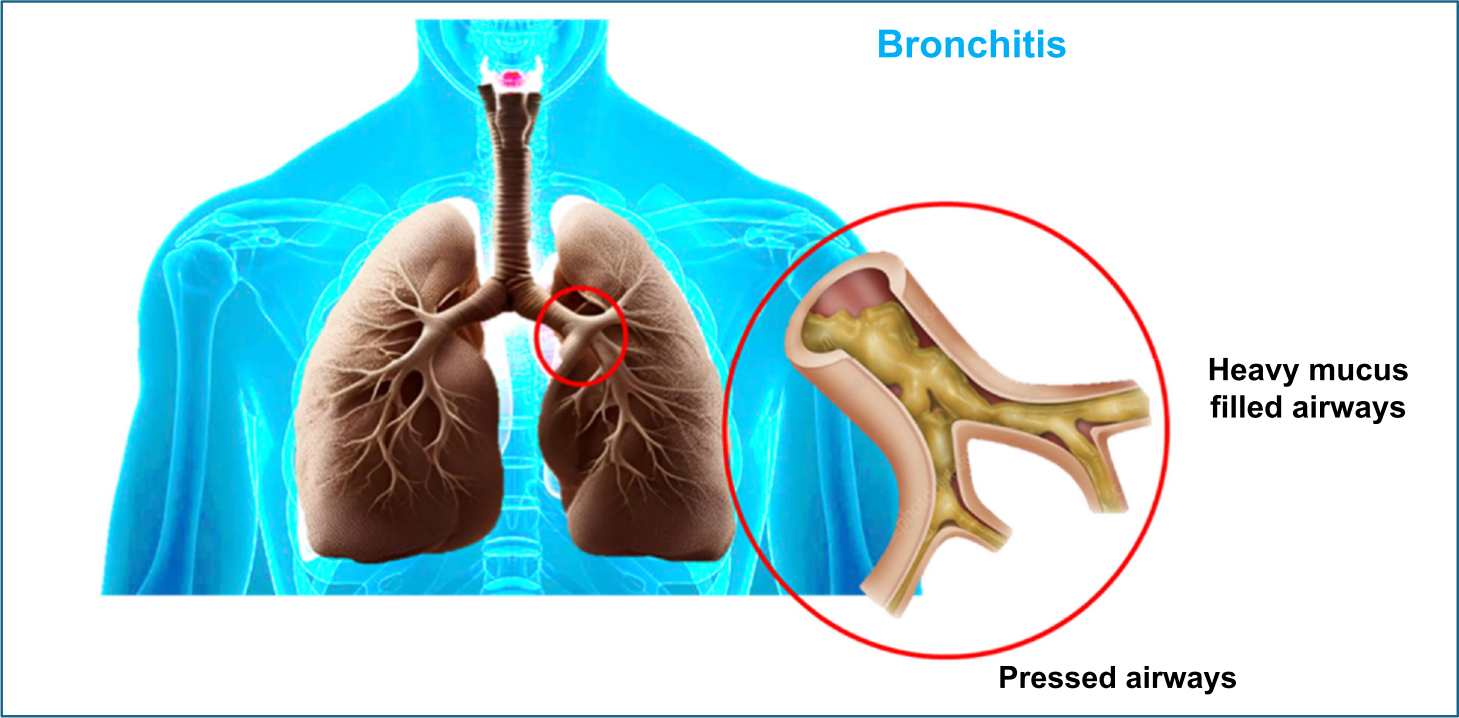
Pressed air passages with heavy mucus in the lungs causing bronchitis.

Long-term lung problems, such as chronic bronchitis, are common in people who smoke [79]. This condition causes ongoing lung inflammation that does not go away. The lungs produce extra mucus, which can block the airways and make breathing difficult [80]. The buildup of white blood cells and swelling in the lungs also harms the tiny air sacs that help us breathe [81] (Fig. **8**).

For a long time, people have used plants as natural medicines. Tulsi (also called Ocimum sanctum) is a common remedy for bronchitis and asthma. Its key ingredient, Eugenol, helps fight germs, reduce pain, relax muscles, and ease stress. Studies show that people with bronchitis recover faster and have fewer symptoms when treated with thyme extract and primrose root. Ivy (Hedera helix) extract has also been found to help with chronic bronchitis. Black cumin (Nigella sativa) contains a substance called Nigellone, which helps relax muscle spasms, but another ingredient, thymoquinone, does not seem to help with asthma [82, 83].

Other plants that might be useful for treating chronic bronchitis include okra (*Abelmoschus esculentus*), Bansa (*Adhatoda vasica*), Indian licorice (*Abrus precatorius*), barkcloth tree (*Antiaris toxicaria*), maidenhair fern (*Adiantum capillus-veneris*), grains of paradise (*Aframomum melegueta*), chickweed (*Ageratum conyzoides*), wild onion (*Allium ascalonicum*), garlic (*Allium sativum*), spiny amaranth (*Amaranthus spinosus*), marshmallow (*Althaea officinalis*), cashew apple (*Anacardium occidentale*), pineapple (*Ananas comosus*), and kirayat (*Andrographis paniculata*), among others. These plants have shown promise in studies for managing bronchitis symptoms [84, 85].

#### 1.2.5 Cystic Fibrosis

Pseudomonas aeruginosa is a major germ that causes long-term lung issues, especially in people with cystic fibrosis (CF) [86]. This bacteria makes it harder to breathe by causing infections and thick mucus to build up in the lungs [87]. CF happens when there is a change in a specific gene called CFTR. This change disrupts how the body moves salt and water, leading to problems like lung infections [88, 89].

Pseudomonas aeruginosa makes CF worse by producing harmful molecules called free radicals and hiding from the immune system, which normally destroys germs [90]. One of its key tools is an enzyme called exoenzyme, which helps the bacteria survive longer and cause more damage [91]. Scientists also discovered that this germ creates traps made of DNA, proteins, and other materials called NETs (neutrophil extracellular traps). These NETs help the bacteria grow in the lungs and make infections worse [92].

Researchers studied how Pseudomonas aeruginosa affects CF patients and found that it uses NETs to take over the lungs. They also noticed that the bacteria can change shape in sick patients, which might explain why some people get sicker than others with CF [93].

Some natural remedies show promise in treating CF. For example, curcumin, found in turmeric, may help fix the faulty CFTR protein. Other herbs like burdock root, comfrey, elecampane, and lobelia can help reduce mucus, strengthen lung tissue, and improve breathing. Plants like skullcap and turmeric can also reduce inflammation and make it easier to clear the airways. Additionally, herbs such as astragalus, garlic, nettle, and reishi mushroom support the immune system and may help with CF symptoms [94-96].

### 1.3 Infectious Lung Diseases

Many common breathing-related illnesses spread from one person to another in places like schools, workplaces, or homes. When an infected person goes to one of these places, they can pass their germs to others, which is why these illnesses are called contagious. The germs spread through the air or by touching something that an infected person has touched. While it is less likely to catch these viruses outdoors, it is still possible.

#### 1.3.1 Pulmonary Pneumoniae

Pneumonia is a condition where the tiny air sacs in the lungs, called alveoli, become inflamed [97]. It can be caused by different types of germs, including bacteria, viruses, fungi, or cryptococcal organisms. Most pneumonia cases happen when germs that naturally live in the nose and throat spread to the lungs. For healthy people, bacteria like Streptococcus pneumoniae and Haemophilus influenzae are common causes of pneumonia caught in everyday life. However, in hospitals, pneumonia is often caused by tougher, more resistant bacteria such as Klebsiella pneumoniae, Pseudomonas aeruginosa, Staphylococcus aureus, and Escherichia coli [98-101]. In people with weakened immune systems, pneumonia is more likely to be caused by fungi, viruses, or unusual bacteria instead of the common germs.

The body has defenses to protect against lung infections, such as the structure of the upper respiratory tract, the branching airways, mucus that traps germs, tiny hairs (cilia) that push mucus out of the lungs, and the reflex to cough [102].

**Fig. (9):**
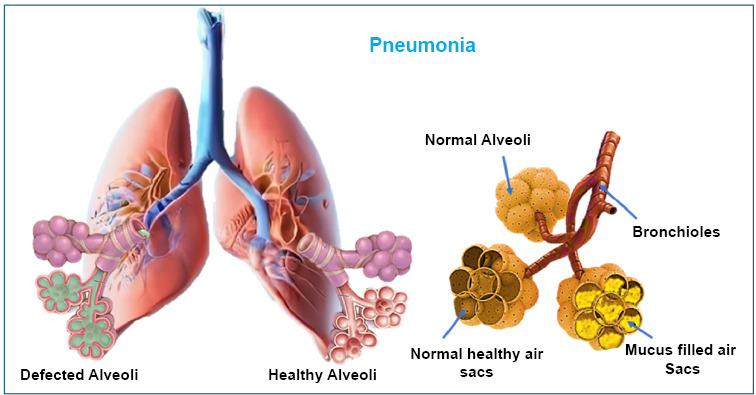
Graphical diagram of pulmonary pneumoniae representing mucus-filled air sacs or alveoli.

Some plants, like those in the Verbascum (mullein) family, have been used in traditional medicine to treat pneumonia because they can fight germs [103]. One study found that an extract from Verbascum fruticulosum was effective against a drug-resistant strain of Streptococcus pneumoniae. Another plant, Urtica urens (dwarf nettle) [104], also reduced the bacteria, but it was not as effective as the Verbascum extract [105, 106] (Fig. **9**).

Beetroot, scientifically known as Beta vulgaris, is a common vegetable eaten around the world. The alcoholic extracts from beetroot leaves, made using substances like n-hexane and chloroform, have shown strong antibacterial effects against Klebsiella pneumoniae, a bacteria that can cause pneumonia. Other plants used to treat pneumonia include Gular fig (Ficus racemosa), Nepeta glutinosa, Castor bean (Ricinus communis), Myrobalan (Terminalia chebula), and Chaste tree (Vitex negundo) [107].

#### 1.3.2 Pulmonary Tuberculosis

Tuberculosis (TB) is a disease caused by a bacterium called Mycobacterium tuberculosis [108]. This bacterium is acid-fast, needs oxygen to grow, and has a rod shape. TB can affect many parts of the body and shows up in different ways. According to the World Health Organization (WHO), TB is the deadliest infectious disease globally, with over 10 million people getting sick and more than 250,000 dying from it every year [109]. TB symptoms often develop slowly over weeks or months, but in young children or people with HIV, symptoms can appear quickly. Common signs include a persistent cough, weakness, fever, weight loss, night sweats, and loss of appetite. In some cases, patients may cough up blood or have difficulty breathing [110].

TB is spread through the air when someone with active TB in their lungs coughs, sneezes, or spits, releasing tiny droplets that carry the bacteria [111]. When another person breathes in these droplets, the bacteria settle in their lungs' air sacs (alveoli). There, the bacteria multiply inside immune cells called macrophages, causing the release of chemicals that attract other immune cells to form a cluster called a granuloma. Granulomas are key in diagnosing TB [112].

Natural remedies, including medicinal plants, have been used to find new and more effective treatments for TB. Plants are valuable because they offer diverse and powerful natural chemicals that can target infections. Many parts of plants, like leaves, roots, and bark, are used to make medicines in the form of teas, tinctures, or infusions. These treatments are part of ancient healing traditions in countries like China, India, Greece, and Egypt. Even today, over half the world’s population relies on medicinal plants for healthcare [113].

Some common plants used to treat TB include Artemisia afra, Lippia javanica, Cannabis sativa, Zingiber officinale (ginger), and Bidens pilosa, among others [114, 115].

### 1.4 Malignant Lung Diseases

#### 1.4.1 Bronchogenic Carcinoma

Lung cancer, also known as bronchogenic carcinoma, occurs when cells in the lungs grow uncontrollably and do not stop dividing. It is one of the leading causes of death worldwide and is the most common and deadly type of cancer [116]. The main challenges in treating lung cancer include diagnosing it late and not having effective treatments. Lung cancer is divided into two main types: small-cell lung cancer (SCLC) and non-small cell lung cancer (NSCLC) [117]. It accounts for 40% of all cancer cases, and by 2020, 2.8 million people had been diagnosed with it [118].

Smoking is the leading risk factor for lung cancer, causing over 80% of cases in men and about 70% of cases in women in Northern Europe, with 45% globally linked to smoking [119, 120]. Other risk factors include exposure to harmful substances like asbestos, radon, chromium VI, nickel, and polluted air.

**Fig. (10):**
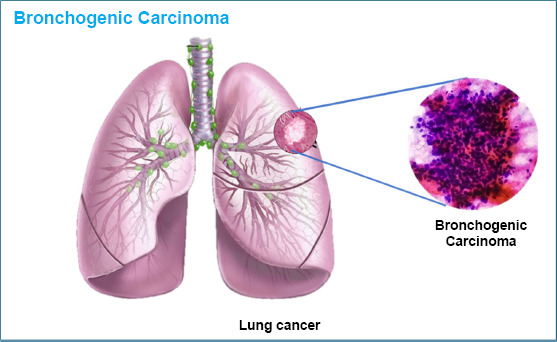
Carcinogenic or mutant cells over lung surface in bronchogenic carcinoma.

Moreover, to address these challenges, natural compounds have gained attention as alternatives to traditional treatments because they tend to have fewer side effects [121]. Cancer cells often become resistant to chemotherapy and continue to spread. However, natural substances like phytochemicals show promise because they can kill cancer cells, reduce inflammation, and fight oxidative stress. Compounds like flavonoids, phenols, alkaloids, and carotenoids have been studied for their ability to trigger cancer cell death, stop the spread of tumors, and prevent the growth of blood vessels that tumors need to survive [122]. Combining these natural compounds with chemotherapy may enhance treatment effectiveness by targeting cancer cells and slowing their growth (Fig. **10**).

Phyto-treatment uses various plants and herbs to treat diseases. Some examples include Selaginella tamariscina, saffron (*Crocus sativus*), Toona sinensis, Sesbania grandiflora, Descurainia sophia, ginseng (*Panax ginseng*), ginger (*Zingiber officinale*), Embelia ribes, Salvia miltiorrhiza, curry leaves (*Murraya koenigii*), Lysimachia capillipes, Scutellaria baicalensis, cinnamon (*Cinnamomum subavenium*), and Davallia divaricata. Other herbs used to treat bronchogenic carcinoma (a type of lung cancer) include *Daphne genkwa*, *Curcuma wenyujin*, *Polygala senega*, *Croton macrostachys*, *Cassia garrettiana*, and *Brucea javanica* [123, 124]. Refer to Table **1** below.

## CONCLUSION

Plants with natural healing properties are being studied to create treatments for diseases. Examples include substances like curcumin, gingerols, and zingerone, which come from plants. While these show potential, more detailed studies are needed to confirm their safety and effectiveness for medical use. This review looks at medicinal plants and how they might help treat lung conditions. These plants have compounds with helpful effects, such as reducing inflammation, fighting infections, and supporting the immune system. As a result, herbal medicine could be a good alternative to regular medications, often with fewer side effects. Even though people have used herbal remedies for respiratory issues for a long time, more research is needed to fully understand how they work.

## Figures and Tables

**Table 1:** Natural herbs and plants used in the treatment of respiratory diseases.

**Type of Pulmonary Disease**	**Pulmonary Disease Indications**	**Medicinal Herbal Treatments**	**References**
**Idiopathic pulmonary fibrosis**	Breathlessness; cough without moisture excessive fatigue; unintended weight loss; aching joints and muscles; Widening and rounding of the tips of the fingers or toes	Pippali *Cordyceps sinensis* *Astragaloside* *Salviae Miltiorrhizae* *Isoliensinine* Triptolides Curcumin	[125, 126]
**Pulmonary sarcoidosis**	Dry cough that never goes away; breathlessness; sighing; chest pain; enlarged lymph nodes	*Tribulus terrestris* *Ocimum tenuiflorum* *Asparagus racemosus* *Curcuma longa* *Commiphora wightii* *Phyllanthus emblica* *Withania somnifera*	[107, 127]
**Systemic sclerosis (scleroderma)**	Breathlessness; reduced capacity to tolerate exercise; discomfort; lung tissue damage; regurgitation; trouble swallowing; bloating;	*Capparis spinosa* *Ginkgo biloba* *Cinnamomum cassia* *Wolfiporia extensa* *Paeonia suffruticosa* *Paeonia lactiflora* *Paeonia veitchii* *Prunus persica* *Prunus davidiana* *Oenothera biennis* *Tripterygium wilfordii* *Bupleurum chinense* *Paeonia lactiflora* *Cyathula officinalis*	[48, 128]
**Chronic obstructive pulmonary disease**	Breathing problems; chest tightness; persistent cough with clear, white, yellow, or greenish discharge; reoccurring respiratory tract infections; lack of energy; edema in the ankles, legs, or feet	*Acacia leucophloea* *Tussilago farfara* *Chelidonium majus* *Seutellaria baicalenei* *Datura stramonium* *Epihedra sinica* *Hederae helices* *Eugenia caryophyllata* *Sida cordifolia* *Symphytum officinale*	[129, 130]
**Pulmonary arterial hypertension**	Heightened dyspnea; swelling in the legs, feet, neck and abdomen; dizziness and moments of fainting; chest ache; palpitations of the heart; fingers and lips going blue;	*Allium macrostemon* *Allium sativum L.* *Allium ursinum L.* *Crataegus rhipidophylla* *Crataegus rhipidophylla* *Eulophia macrobulbon* *Kelussia odoratissima* *Mimosa pigra L.* *Moringa oleifera* *Rhodiola tangutica* *Salvia miltiorrhiza* *Salvia miltiorrhiza* *Salvia miltiorrhiza* *Securigera securidaca* *Terminalia arjuna* *Trifolium pretense L.* *Withania somnifera*	[69, 70]
**Asthma**	Loss of breath while speaking, eating, or sleeping; tightness in the chest that persists and gets worse; accelerated heart rate; tiredness, confusion, and drowsiness; fingers or lips turning blue	*Agastache mexicana* *Ammi visnaga* *Astragalus membranaceus* *Coffea arabiga* *Echinacea purpurea* *Emblica officinalis* *Ephedra sinica* *Ephedra trifurca* *Foeniculum vulgare* *Ginkgo biloba* *Lippia dulcis* *Lobelia inflata* *Mentha pulegium* *Talauma mexicana* *Thea sinensis* *Theobroma cacao* *Tussilago farfara* *Verbascum thapsus*	[76, 131, 132]
**Bronchitis**	Cough; mucus production stained with blood; breathlessness; mild fever and chills; discomfort in the chest	*Acalypha indica* *Acacia torta* *Lactuca virosa* *Ocimum sanctum* *Convolvulus pluricaulis* *Mentha haplocalyx*	[133, 134]
**Cystic fibrosis**	Periodic lung infections; frequent pneumonia; breathing difficulties: persistent coughing	*Astragalus membranaceus* *Galium aparine* *Allium sativum* *Fucus vesiculosus* *Urtica diotica* *Ganoderma lucidum* *Echinacea purpurea* *Hydrastis canadensis* *Baptisia australis*	[95, 107]
**Pneumonia**	Chest pain during respiration; frequent dizziness; mucus rich coughing; fatigue; fever and periodic body chills; diarrhea, vomiting or nausea; breathlessness	*Justicia adhatoda* *Achyranthes aspera* *Mangifera indica* *Foeniculum vulgare* *Phoenix dactylifera* *Cordia obliqua* *Onosma bracteatum* *Glycyrrhiza glabra* *Thymus vulgaris* *Mentha piperita* *Hyssopus officinalis* *Abelmoschus esculentus* *Acacia arabica* *Ficus religiosa* *Ziziphus vulgaris* *Withania somnifera* *Phyla nodiflor* *Viola odorata* *Curcuma longa* *Zingiber officinale*	[135, 136]
**Pulmonary tuberculosis**	Breathing difficulty; coughing up blood; chest pain; cough (usually with mucus); fever; weight loss; excessive sweating, particularly at night; fatigue; wheezing	*Cycas circinalis* *Orchis mascula* *Eclipta alba* *Pistacia lentiscus* *Tephrosia purpurea* *Curcuma longa* *Picrrohiza kurooa* *Phyllanthus amarus* *Pinius succinifera*	[115]
**Bronchogenic carcinoma**	Cough that worsens; blood-filled mucus; chest pain during activity; distorted voice; reduced appetite; breathlessness;	*Perilla frutescens* *Platycodon grandiflorum* *Tussilago farfara* *Prunus armeniaca* *Rhus verniciflua* *Stemona japonica* *Draba nemorosa* *Morus alba*	[124, 137]

## LIST OF ABBREVIATIONS

COPD: = Chronic Obstructive Pulmonary Disease
RLDs: = Restrictive Lung Diseases
CF: = Cystic Fibrosis
SCLC: = Small-cell Lung Cancer
NSCLC: = Non-small Cell Lung Cancer
